# Bragg coherent diffraction imaging and metrics for radiation damage in protein micro-crystallography

**DOI:** 10.1107/S1600577516017525

**Published:** 2017-01-01

**Authors:** H. D. Coughlan, C. Darmanin, H. J. Kirkwood, N. W. Phillips, D. Hoxley, J. N. Clark, D. J. Vine, F. Hofmann, R. J. Harder, E. Maxey, B. Abbey

**Affiliations:** a ARC Centre of Advanced Molecular Imaging, Department of Chemistry and Physics, La Trobe Institute for Molecular Science, La Trobe University, Victoria 3086, Australia; b CSIRO Manufacturing Flagship, Parkville 3052, Australia; c Stanford PULSE Institute, SLAC National Accelerator Laboratory, Menlo Park, CA 94025, USA; d Center for Free-Electron Laser Science (CFEL), Deutsches Elektronensynchrotron (DESY), Notkestrasse 85, 22607 Hamburg, Germany; e Advanced Light Source, Berkeley Lab, Berkeley, CA 94720, USA; f Department of Engineering Science, University of Oxford, Oxford OX1 3PJ, UK; g Advanced Photon Source, Argonne National Laboratory, Argonne, IL 60439, USA

**Keywords:** radiation damage, dose, protein crystallography, micro-crystallography, Bragg coherent diffractive imaging

## Abstract

The proliferation of extremely intense synchrotron sources has enabled ever higher-resolution structures to be obtained using data collected from smaller and often more imperfect biological crystals (Helliwell, 1984 ▸). Synchrotron beamlines now exist that are capable of measuring data from single crystals that are just a few micrometres in size. This provides renewed motivation to study and understand the radiation damage behaviour of small protein crystals. Reciprocal-space mapping and Bragg coherent diffractive imaging experiments have been performed on cryo-cooled microcrystals of hen egg-white lysozyme as they undergo radiation damage. Several well established metrics, such as intensity-loss and lattice expansion, are applied to the diffraction data and the results are compared with several new metrics that can be extracted from the coherent imaging experiments. Individually some of these metrics are inconclusive. However, combining metrics, the results suggest that radiation damage behaviour in protein micro-crystals differs from that of larger protein crystals and may allow them to continue to diffract for longer. A possible mechanism to account for these observations is proposed.

## Abstract 2

A combination of Bragg coherent diffractive imaging and reciprocal-space mapping results indicate that the global radiation damage behaviour of micro-crystals is different compared with macroscale crystals.

## 1. Introduction   

Over the past 50 years macromolecular crystallography has been the primary method for establishing the structure of proteins. Progress with optimizing and improving protein crystallography beamlines has been such that the collection of partial datasets from single crystals that are just a few micrometres in size is now possible. Theoretical predictions based on radiation damage behaviour suggest that a complete dataset could be collected from a protein crystal as small as 1.2 µm (Holton & Frankel, 2010 ▸). In practice though, poor signal-to-noise generally prevents data collection once the diffraction pattern intensity decays by an appreciable amount. Complete datasets from samples this small can be collected however, provided many different crystals are measured in the beam, the quality of individual crystals is sufficient to observe diffraction, and the crystal lattices are sufficiently homogeneous that partial datasets can be merged. Consequently, following successful proof-of-principle experiments, serial synchrotron X-ray crystallography (SSX) is now rapidly becoming established as a viable route to protein structure determination (Stellato *et al.*, 2014 ▸; Gati *et al.*, 2014 ▸).

With the introduction of cryo-cooling in protein crystallography the problem of radiation damage was greatly reduced (Hope, 1988 ▸). However, the collection of X-ray diffraction data with more intense beams and from ever smaller crystals continues to make radiation damage a topic of prime importance for protein crystallography (Murray & Garman, 2002 ▸; Garman & Owen, 2006 ▸). Furthermore, interpreting data collected from samples which have already been damaged can be extremely challenging and has partly motivated extensive efforts in understanding the effects of radiation damage in protein crystallography. Radiation damage can alter the protein structure through bond breaking (commonly di­sulfide bonds or removal of side chains), heating and the creation of free radicals which interact with the protein and cause damage. Whilst it is known that bond breakage (specific chemical damage) within the crystal can occur prior to the diffraction pattern being visibly altered, structural damage in the crystal eventually leads to spot fading and a modification of the measured Bragg peak intensity distribution. Higher-resolution spots generally fade faster than low-resolution peaks, implying that small spatial features are destroyed first by radiation damage, but may also be because of small displacements of the protein within the unit cell or unit cell expansion. At 100 K, the rate of overall loss of resolution, global radiation damage, is essentially the same for every protein and depends on the specific reflection under consideration and its associated *d*-spacing (Holton, 2009 ▸).

For macroscopic crystals the collection of a complete, full dataset from a single-crystal opens up a range of possibilities for analysis of both specific and global radiation damage. Two of the most frequently used parameters for monitoring radiation damage are: total scattered intensity (Teng & Moffat, 2000 ▸; Leal *et al.*, 2013 ▸) and change in full-width half maxima (FWHM) of the peak (Hu *et al.*, 2004 ▸).

**Figure 1:**
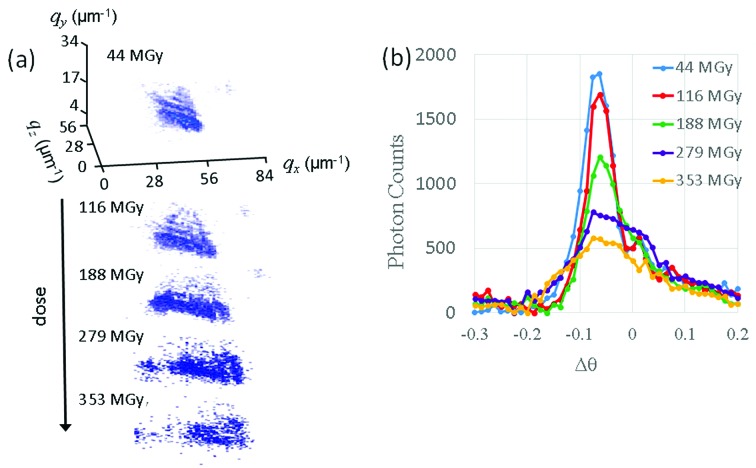
Example dataset collected from a single micrometre-sized HEWL protein crystal at 100 K; (*a*) 3D rendering of the reciprocal-space map as a function of dose and (*b*) the corresponding θ rocking curves.

In the present case, however, extremely small crystals (<2 µm) are being measured and real-space information recovered from the diffracting micrometre-sized crystal as it undergoes radiation damage. This places several constraints on the type of data that can be collected. For example, in order to determine the unit cell volume, normally only a couple of images, generally 90° apart, are required. However, due to the setup used for Bragg coherent diffractive imaging (BCDI) in which only one reflection is measured at a time, combined with the rapid damage of the micrometre-sized crystals measured, determining the unit cell volume was not possible in the current experiment. Three possible metrics for radiation damage that can be extracted from coherent reciprocal-space map data are: integrated single Bragg peak intensity, change in *d*-spacing and the widths of any rocking curves. Fig. 1 ▸ shows a typical example of the data collected at 100 K in a coherent imaging experiment from a protein crystal undergoing radiation damage.

The intensity of individual Bragg peaks depends on a number of parameters including diffracting crystal volume, crystal packing, crystal structure and quality, the time for data collection and the geometry of diffraction (Holton, 2009 ▸). Therefore the exact behaviour of peak intensity with radiation damage varies between different crystals and reflections. The reliability of any conclusions drawn from Bragg intensity data will therefore depend on the knowledge of these factors. In the experiment performed here, the full spot intensity is obtained by integrating over the Bragg peak as it moves through the Ewald sphere during rotation of the crystal in and out of the diffraction condition. The reflections studied all appear at similar Bragg spacings such that, for *relative* measurements of the integrated intensity of individual Bragg reflections, the dependence on the geometry of diffraction should be similar for all datasets. Any significant differences in the data are therefore likely to be attributed to variations in the individual crystal parameters rather than to differences in the measurement. Although a detailed understanding of the underlying mechanisms for radiation damage is still an active area of research, on the basis of experimental observations, models for average spot intensity tend to find that intensity at a given resolution fades exponentially at all temperatures (Holton, 2009 ▸).

Unit cell expansion has also been discussed extensively in the literature in the context of radiation damage; however, a number of authors have pointed out that the unit cell is an unreliable metric for radiation damage. Both room-temperature (Southworth-Davies *et al.*, 2007 ▸) and cryo-crystallography studies conducted at 100 K (Murray & Garman, 2002 ▸; Ravelli *et al.*, 2002 ▸) have shown that the rate of change of the unit cell varies for different proteins. In the present case, the behaviour of the lattice spacing, *d*, as a function of absorbed dose can be examined. Although not a direct measure of the change in unit cell volume, *d*-spacing can be used to qualitatively compare the behaviour of these hen egg-white lysozyme (HEWL) micro-crystals with similar studies reporting unit cell volume changes in the literature.

The FWHM of the Bragg peak rocking curve is another quantity analysed here that has previously been examined as a function of radiation damage. Although the rocking curve widths are a convolution of lattice change and non-uniform illumination, in the experiments described here it is assumed that the crystal illumination is constant as the crystal undergoes radiation damage. Therefore the change in the rocking curve width is interpreted as a relative change, which can be directly related to the radiation damage. Through measurements of the rocking curve profile, mosaicity and elastic strain have been characterized in both room temperature and cryo-cooled protein crystals of HEWL (Hu *et al.*, 2004 ▸; Lovelace *et al.*, 2006 ▸). For example, broadening of the rocking curves collected from tetragonal HEWL crystals of a few percent was observed by Hu *et al.* (2004 ▸) during illumination by X-rays.

Although the field of radiation damage in protein crystallography is very well established, the extension of many of these ideas to micrometre-sized crystals is still an area of active research (Ziaja *et al.*, 2012 ▸). For example, exponential decay trends have been established in the behaviour of Bragg peak intensity as a function of dose for large crystals (Wang & Ealick, 2004 ▸; Diederichs, 2006 ▸; Diederichs *et al.*, 2003 ▸; Ravelli *et al.*, 2003 ▸; Holton, 2009 ▸), but it is unclear whether the same trends would persist for micrometre-sized crystals. Hence, radiation damage in protein micro-crystallography is an issue which needs to be addressed in order to plan experiments but also to correctly interpret the data.

In this work, high-resolution Bragg peak images are recorded on an area detector whilst rocking the crystal in and out of the Bragg condition. The rocking curve profiles can be studied along any direction in reciprocal space as well as examining the overall reciprocal-space map (RSM) (3D Bragg intensity distribution) volume changes during radiation damage. A critical difference here compared with previous work in analysing rocking curves to study crystal perfection and radiation damage is that the evidence from the present experiments suggests that the rocking curve width in the present case is dominated by the shape function of the crystal. Previous reports in the literature have looked at crystals orders of magnitude larger than the ones studied here, and thus convolution with the crystals shape function causes minimal peak broadening.

Previous radiation damage investigations for micrometre-sized protein crystals have also indicated that, for an isolated crystal, the radiation damage behaviour may be different compared with larger crystals (Sanishvili *et al.*, 2011 ▸). One explanation for this is that, once the dimensions of the crystal become smaller than or comparable with the primary photoelectron mean free path, radiation damage may be mitigated (Stern *et al.*, 2009 ▸; Nave & Hill, 2005 ▸; Moukhametzianov *et al.*, 2008 ▸). A similar line of reasoning is given in published reports showing that the use of micrometre-sized beams with large crystals reduces the rate of radiation damage (Sanishvili *et al.*, 2011 ▸; Finfrock *et al.*, 2010 ▸, 2013 ▸). For example, Finfrock *et al.* have specifically discussed the spatial dependence of dose using a line-focused microbeam showing that, for the 18.6 keV X-rays used in their experiment, at around 1 µm there is a region of higher deposited energy. Although the data in that paper [specifically Fig. 5 in Finfrock *et al.* (2010 ▸)] need to be adjusted to account for the lower X-ray energy and actual beam size used here, this offers a possible route to interpreting data obtained in the present experiments. The same reduction in radiation damage is not necessarily expected, however, if the volume illuminated includes the surrounding cryo-protected solvent. This is because the photoelectrons generated in this solvent deposit energy in the crystal *via* secondary effects.

In this present study the BCDI technique is used to study radiation damage effects in micrometre-sized protein crystals. BCDI involves iteratively recovering the phase of the scattered intensity associated with individual Bragg reflections, enabling a direct transform and recovery of an image of the crystal (Robinson & Vartanyants, 2001 ▸). For a coherently illuminated crystal, the continuous diffracted intensity around each Bragg reflection is described by (Robinson & Vartanyants, 2001 ▸; Abbey, 2013 ▸)
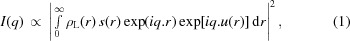
where 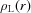 is the electron density of the crystal, 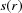 the shape function describing the diffracting volume, 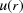 the relative displacements of the atoms from their ideal lattice positions, and *r* and *q* are position vectors in real and reciprocal space, respectively. The use of CDI in the Bragg geometry (BCDI) is now a well established method for characterizing micro- and nanocrystals of small molecule materials science samples (Newton *et al.*, 2010 ▸; Pfeifer *et al.*, 2006 ▸). However, largely due to the difficulties with applying this technique to radiation-sensitive samples, there have been relatively few reports of BCDI being applied to protein crystals. The first such report was by Boutet *et al.* who used the technique to study the collapse of the holoferritin crystal lattice due to radiation damage (Boutet & Robinson, 2008 ▸). This initial demonstration was followed up much later by Coughlan *et al.* who used BCDI to study a single HEWL crystal in both 2D and 3D (Coughlan *et al.*, 2015 ▸, 2016 ▸). A detailed analysis of the coherent diffraction patterns collected from polyhedrin crystals was recently performed by Nave *et al.* who also examined the phase information retrieved using BCDI to look at the crystal imperfections at sub-micrometre spatial resolution (Nave *et al.*, 2016 ▸).

Although when using the BCDI approach there is no measurement of information about the evolution of the molecular structure, the technique allows deconvolution of real- and reciprocal-space information. This provides a new window into the global damage effects allowing the evolution of the average crystal properties to be seen directly at nanometre resolution whilst radiation damage is occurring. In the current work, images of the crystals real-space electron density have been examined as a function of radiation damage and this information has been compared with more well established radiation damage metrics. It should be noted that the discussion here is mainly restricted to the electron density (amplitude) image information rather than the disorder (phase) information. This is mainly because for the six HEWL crystals studied here the phase showed little internal structure. This could be due to the fact that the crystal quality was very good or perhaps the image resolution (tens of nanometres) was insufficient to resolve small internal variations in the lattice quality. As demonstrated by Nave *et al.* (2016 ▸) though, for some samples a great deal of additional useful information about defect density, mosacity *etc*. can be obtained from the phase information provided by CDI.

In the present set of experiments both micrometre-sized protein crystals and a beam that was slightly larger but comparable with the size of the crystal were used. The results suggest that in this case radiation damage is reduced, even though the crystal is embedded in cryo-protectant and fully illuminated by the micro-focused beam.

The key elements of the present study in comparison with previous radiation damage studies in this area may be summarized as follows:

(i) For six, micrometre-sized, cryo-cooled HEWL crystals the variation in the intensity, relative *d*-spacing and rocking curve width are compared; these are established metrics for radiation damage within the literature.

(ii) The volume of the RSM and real-space images associated with these crystals obtained through coherent imaging are investigated as possible additional radiation damage metrics for micrometre-sized crystals.

Based on the collective observations, the radiation damage behaviour of micrometre-sized (in the present case <2 µm) protein crystals is compared and contrasted with that of larger, macroscopic crystals reported in the literature.

## 2. Materials and methods   

### 2.1. Sample preparation and characterization   

Oversampled BCDI data were collected at the Advanced Photon Source (APS) on beamline 34-ID-C; procedures for sample preparation and data collection are described in a previous paper applying BCDI to HEWL crystals (Coughlan *et al.*, 2016 ▸). Briefly, tetragonal (*a* = *b* = 79 Å, *c* = 37 Å, α = β = γ = 90°, *P*4_3_2_1_2) micrometre-sized HEWL crystals were grown using the batch method. 40 mg ml^−1^ of protein in 0.5 *M* acetic acid buffer (pH 4) was mixed with precipitant buffer [6% PEG 6 K and 18%(*w*/*v*) NaCl at pH 4] at a ratio of 1:3.5 protein to precipitant. The preparation was optimized to produce micrometre-sized crystals of HEWL with a narrow size distribution. Samples were characterized following previously published protocols (Coughlan *et al.*, 2016 ▸, 2015 ▸; Darmanin *et al.*, 2016 ▸). An Olympus BX optical microscope was used to image the crystals. Although the size of the crystals was at the resolution limit, the optical microscope enables a large number of crystals to be imaged quickly, providing a good sampling of the overall size distribution. Electron microscopy was used to obtain a more accurate estimate for the size of a small number of representative crystals. Transmission electron microscope (TEM) measurements were made using a Jeol JEM-2010 TEM operated at 100 keV. Images were taken using a Valeta 4 MP CCD camera. TEM images were analysed using *IMAGEJ* (Schneider *et al.*, 2012 ▸), an open source scientific image analysis software package. The average and standard deviation for the crystal size was calculated as 1.27 ± 0.44 µm by 1.16 ± 0.40 µm. Optical micrographs and TEM images of lysozyme microcrystals prepared under identical conditions to those described here are given by Coughlan *et al.* (2016 ▸) and Darmanin *et al.* (2016 ▸). Using this protocol no aggregation of crystals was observed.

### 2.2. Data collection and RSM analysis   

For data collection, the samples were mounted onto MicroMeshes MiTeGen crystallography loops (400/10 mesh) containing either 50% polyethyl­ene glycol (PEG) 400 or glycerol cryo-protectant. Prior to mounting the samples they were plunge cryo-cooled in liquid nitro­gen. An Oxford Instruments Cryojet which produced 100 K gaseous nitro­gen was set up above the sample stage. The loop was mounted on a goniometer stage and data collected using 9 keV (0.1378 nm) X-rays focused to a spot size measuring 1.7 µm by 1.3 µm FWHM with a total flux of 5 × 10^9^ photons s^−1^. The Medipix2 photon-counting detector which was used had 256 × 256 square pixels of 55 µm side length and was mounted on a diffractometer arm perpendicular to the scattering vector; an evacuated flight tube was installed to minimize air scatter along the flight path. Here three rotation angles are defined: θ corresponds to rotation about the vertical axis, χ to rotations about the incident beam and φ to rotations in the direction of the incident beam vector, perpendicular to the other two rotation axes. For the RSM data, rocking curves were measured by placing the detector 1.7 m from the sample at the centre of the Bragg peak and rocking the sample in the θ direction through a total angular range of approximately 0.4°. The step size for the rocking curve was 0.01° with an exposure time of 5 s per step. This measurement was repeated until the Bragg peak intensity dropped below the background threshold of 1 photon measured on the Medipix2 detector. A detailed schematic of the experimental set-up including the coordinate system is given by Coughlan *et al.* (2015 ▸). Due to the high doses imparted to the sample, matrix deformation leading to parasitic crystal rotations can occur, which causes instabilities in the Bragg reflection. For example, whilst aligned to the peak of the rocking curve, the reflection might move out of the Bragg condition or the centre of mass of the reflection on the detector may change. In practice, the effects of matrix deformation are quite obvious and the associated data can readily be excluded from any further analysis.

In addition to rejecting data on the basis of instabilities introduced by matrix deformation, data were selected according to the quality and reliability of the BCDI reconstructions. From a random start, multiple reconstructions were repeated and only those that showed reliable and consistent convergence to the same reconstructed image were retained. The process of rejecting datasets on the basis of instabilities and lack of convergence of the BCDI reconstruction left the six datasets which are analysed in this paper.

The intensity was calculated for each Bragg peak by integrating across the entire 3D peak. As the radiation damage rate has a resolution dependence, the *d*-spacings were checked for each reflection to ensure that they remained within approximately the same volume of reciprocal space. To within the angular resolution of the diffractometer, all the data, which could potentially originate from different *hkl* values, were collected between 13 Å and 17 Å resolution. Due to the geometrical constraints of the BCDI set-up and the limited volume of reciprocal space measured, it was problematic to determine the exact position of the incident beam vector. Hence for many reflections it was not possible to measure the *d*-spacing to sufficient accuracy for precise indexing. However, the 2θ values can be estimated for the reflections measured.

The FWHM of the Bragg peak was calculated along the three orthogonal directions in reciprocal space (*q*_*x*_, *q*_*y*_ and *q*_*z*_). This was achieved by summing the intensity in 2D slices and plotting this as a function of their respective angular values. Typically, only the rocking curve as a function of θ is analysed in the literature (Lübbert *et al.*, 2004 ▸). The spacing of the fringes, which were present in all of the RSM data presented here, can be used to estimate the crystal size for a limited number of projections through the crystal. Measurements of the fringe spacing were used to check that the size of the crystals (at the start of the damage measurements) were consistent with the size distribution obtained *via* TEM analysis (Coughlan *et al.*, 2016 ▸). To estimate the total volume of the RSM, which is sensitive to any changes in the crystal shape function as well as to the formation of crystal mosaic blocks, elastic strain and defect density, the number of voxels with a value above 1 photon was calculated.

The change in *d*-spacing for the micrometre-sized crystals was determined by measuring the shift of the centre of mass of the peak in reciprocal space for each 3D Bragg peak measurement. This shift was then interpreted as a change in the lattice spacing of the crystal due to radiation damage. The conversion between the detector pixel size and the reciprocal-space pixel size was determined by
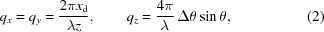
where *x*_d_ is the detector pixel size, Δθ is the rocking curve angular increment (0.1°) and *z* is the detector-to-sample distance. The equivalent change in *d*-spacing is then determined from (Müller *et al.*, 2002 ▸)
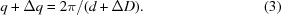



### 2.3. Bragg coherent diffractive imaging reconstructions and analysis   

In principal, once the phases have been correctly assigned to the measured diffracted intensities it is possible to perform a 3D Fourier transform to recover a 3D image of the crystal. However, in practice the 3D reconstruction was only successful in a limited number of cases where the time to collect a complete rocking curve was much shorter than the time taken for significant radiation damage to occur. The 3D reconstruction of a protein crystal using BCDI is discussed by Coughlan *et al.* (2016 ▸). Reconstruction of 2D projections of the protein micro-crystal from single points on the rocking curve are, however, much easier to achieve since individual reconstructions are from data collected in seconds rather than minutes. Another key point is that the crystal sizes used for the present experiments are comparable with the coherence length at beamline 34-ID-C (Huang *et al.*, 2012 ▸; Leake *et al.*, 2009 ▸). To characterize and account for the effects of partial coherence the fringe visibility (which was typically >80%) was checked and an algorithm developed by Clark *et al.* (2012 ▸) was used, which incorporates partial coherence in the image reconstruction process (Chen *et al.*, 2012 ▸; Clark *et al.*, 2012 ▸).

For the 2D real-space images presented and analysed in this paper, data collected at the peak of the rocking curve in the θ direction were used. To reconstruct these images, a combination of error reduction (ER) and hybrid input–output (HIO) algorithms were used. Each reconstruction is the result of averaging 30 independent reconstructions of 4000 iterations generated from a random phase starting guess. Each independent reconstruction contained 210 HIO iterations in blocks of 30 at 100, 500, 800, 1000, 1500, 2000 and 3000 iterations of ER. The 2D support for the crystal was fixed to be a square of 2.2 µm × 2.2 µm; no refinement of the support during the reconstruction was required.

In spite of careful alignment of each crystal to the beam position, there is a risk that the crystal could move out of the illumination volume during the rocking curve. In the few cases where this happened the results were obvious: the intensity of the reflection would fall off immediately (rather than more slowly due to damage). In addition, any fringes around the central Bragg peak would rapidly disappear and the peak intensity would appear at a significantly different 2θ. Another indication of beam–sample misalignment is that the BCDI reconstructions fail to produce an image in these cases due to the soft edges of the beam profile. In this paper data are only presented for crystals which, based on these observations, were fully contained within the beam at all times. Consequently, BCDI can successfully be applied to all of the six datasets analysed.

### 2.4. Dose calculations   

Two methods were used to estimate the absorbed dose for the BCDI experiments. For the first method the program *RADDOSE-3D* was used with a default crystal density of 1.2 g ml^−1^ (Murray *et al.*, 2004 ▸; Zeldin *et al.*, 2013 ▸). *RADDOSE-3D* used the inputs of incident flux (5 × 10^9^ photons s^−1^) (http://www.aps.anl.gov/Beamlines/Directory/showbeamline.php?beamline_id=42), X-ray energy (9 keV), beam size, wedge and crystal size. The ‘calculated dose’ rate for a 1.27 × 1.27 × 1.16 µm crystal was 0.417 MGy s^−1^. However, it should be noted that there are experimental uncertainties associated with the incident beamline flux as well as uncertainties in the size of the crystal which will impact the calculated dose. Full details of how the dose was estimated are given by Coughlan *et al.* (2016 ▸). In calculating the dose, the diffracting volume changes due to damage have been neglected since even attempting to estimate the dose for the part of the crystal which still diffracts would be extremely difficult.

To model the integrated intensity for individual Bragg peaks as a function of dose, the following exponential decay formula (Holton, 2009 ▸) was used,
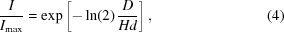
where *I* is the integrated intensity of the RSM after absorbing a dose *D* (MGy), *I*_max_ is the maximum integrated RSM intensity, *H* is the Howells criterion which is usually given as 10 MGy Å^−1^ (*i.e.* loss of 1 Å diffraction resolution for every 10 MGy absorbed dose) and *d* the lattice spacing in Å. Using this formula and the calculated dose rate (*RADDOSE-3D*), the value of *H* which gave the best fit to the measured intensity data for each crystal was determined.

## 3. Results and discussion   

Radiation damage in micrometre-sized protein crystals illuminated by a beam with a FWHM only slightly larger than the crystal was investigated using RSM and BCDI. The relative change in intensity, *d*-spacing, FWHM of the rocking curves and change in RSM volume were recorded as a function of the absorbed dose for six micrometre-sized HEWL crystals. In addition, from the Bragg CDI, complementary real-space information is obtained about the area of the crystal contributing to the diffracted signal.

### 3.1. Integrated RSM intensity   

**Figure 2:**
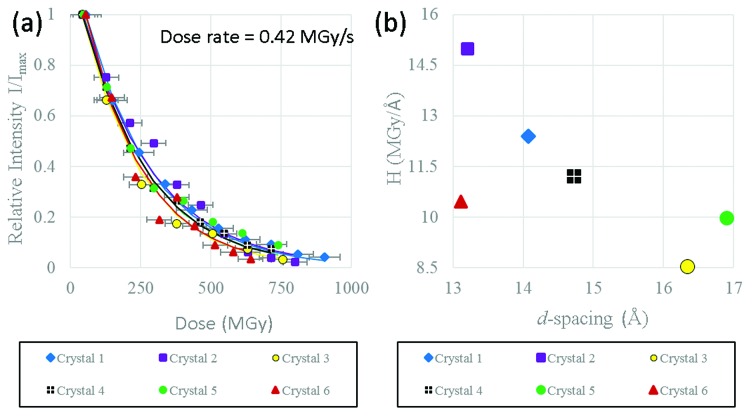
(*a*) Ratio of integrated RSM intensity (*I*) to integrated intensity in the first measured RSM (*I*_max_) as a function of dose. (*b*) Howells criterion determined from least-squares fitting using equation (4) keeping the dose rate fixed to the value calculated from *RADDOSE-3D* (0.42 MGy s^−1^), plotted against the experimentally determined *d*-spacing.

The results for the integrated RSM intensity (Fig. 2 ▸) show very good consistency between datasets collected from different HEWL micro-crystals. The exponential decay of the single Bragg reflection which is observed for every crystal describes the average intensity loss of the Bragg peak which characterizes the global damage as discussed by Holton, who observed the same behaviour at all temperatures (Holton, 2009 ▸). The relative intensity, defined as the ratio of the current integrated intensity to the initial integrated intensity from the first RSM in the time series (*I*_max_), could be matched extremely well using the exponential decay curve of equation (4). This same general trend is also observed in macroscopic crystals at room temperature (Southworth-Davies *et al.*, 2007 ▸) and has been discussed by Holton & Frankel in 2010 (Holton & Frankel, 2010 ▸) where they modelled radiation damage in a number of different crystals at cryogenic temperatures and show the same exponential decay behaviour for each sample. Each data point in Fig. 2(*a*) ▸ requires that a full RSM is collected which takes a finite amount of time, thus the first data points do not start at 0 s. Data are plotted on the *x*-axis at the times at which the peak of the rocking curve is reached, and the horizontal error bars indicate the total time taken to complete each rocking curve.

A least-squares fit of equation (4) to the measured data was used to determine the Howells parameter *H* (which characterizes the sensitivity). Since data for the different crystals were measured under nominally identical conditions it is assumed that the dose rate is the same in each case. However, it should be noted that variations of the crystal size within the beam and the neglect of the influence of photoelectron escape in the calculations means that experimentally some differences in the actual dose may occur even though the setup did not change. The dose rate used for determining *H* was 0.42 MGy s^−1^ calculated in *RADDOSE-3D* using the ‘Gaussian’ model option.

The values for *H* determined from least-squares fitting to the experimental data are shown in Fig. 2(*b*) ▸. Due to the scatter in the data it is difficult to draw firm conclusions about whether there is any relationship between *H* and the *d*-spacing, though it does appear that lower values of *H* may occur at higher *d*-spacing. Note that in general the values determined for *H* are above that of the nominal value of 10 MGy Å^−1^, indicating that the intensity drop-off is slower than would normally be predicted. One possible reason for this is the escape of the photoelectrons which is expected to reduce the rate of intensity decay. Note also that, for the very high doses used here, many of the processes associated with radiation damage will saturate which may alter the value for *H*. From Fig. 2(*a*) ▸ the summed intensity for the six HEWL micro-crystals dropped to 0.7*I*_max_ at a dose of 84 ± 11 MGy (where linear interpolation between nearest-neighbour data points was used and the error quoted is the standard deviation for the six values). This is significantly larger than the absorbed dose limit of 30 MGy (Garman, 2010 ▸; Owen *et al.*, 2006 ▸), though it is important to note that 30 MGy is an experimental limit and not all crystals will tolerate this. However, our data extend well beyond this, up to doses in excess of 800 MGy (reached after 1900 s), a damage regime which has not been well studied in the literature.

### 3.2. Relative *d*-spacing   

**Figure 3:**
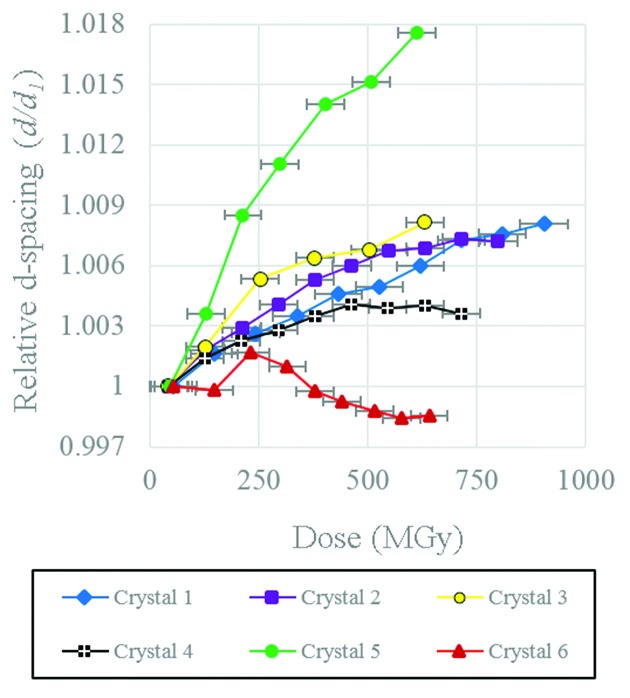
Relative change in *d*-spacing for the six different HEWL crystals. Values were determined from a linear interpolation between the two nearest neighbour data points.

The relative *d*-spacing determined by tracking the centre of mass of the RSM is shown in Fig. 3 ▸ for the individual Bragg peaks moving in 3D reciprocal space. Movement of the centre of mass of the single measured Bragg reflection for each crystal was converted to a change in *d*-spacing according to equations (2) and (3). The variability of the *d*-spacing between crystals was larger than for the corresponding intensity data; however, for five out of the six crystals the *d*-spacing is observed to increase with increasing time/absorbed dose.

For crystals 1 to 5, the average increase in *d*-spacing was 0.89% before the intensity of the reflection dropped below the background threshold value, and 0.39% at the point where the intensity dropped to half its maximum value (*I*_0.5_). However, for crystal 6, after an initial small increase of 0.01% at *I*_0.5,_, the *d*-spacing actually decreased by a total of 0.14%. In general, the behaviour of the relative *d*-spacing as a function of dose appears less consistent between crystals than the integrated intensity loss.

Interestingly, between 0 and 500 s (210 MGy) exposure time, the variation of *d*-spacing for crystals 1 to 5 is linear, in line with results reported for unit cell expansion in the literature (Müller *et al.*, 2002 ▸). However, the majority of previous studies have not investigated the much higher doses examined here for micrometre-sized crystals. In the data, for the majority of crystals (with the exception of crystal 6) the general behaviour is best described by a logarithmic curve. On the basis of the expansion of the *d*-spacing the data indicate that the rate of damage in fact slows after a certain dose, although it should be emphasized that the dependence of *d*-spacing on radiation damage is highly complex and that the observed trends may well be different for different reflections. In the context of protein micro-crystallography this type of behaviour does not appear to have been reported previously. In general, radiation studies on macroscopic protein crystals in relation to the expansion of *d*-spacing with dose was found to follow a linear relationship (Müller *et al.*, 2002 ▸; Ravelli *et al.*, 2002 ▸; Ravelli & McSweeney, 2000 ▸; Murray & Garman, 2002 ▸) or an exponential relationship (Shimizu *et al.*, 2007 ▸). The variability of the *d*-spacing expansion between crystals makes this finding on the basis of the *d*-spacing alone inconclusive, nonetheless the results are intriguing.

### 3.3. Relative FWHM and RSM volume change   

**Figure 4:**
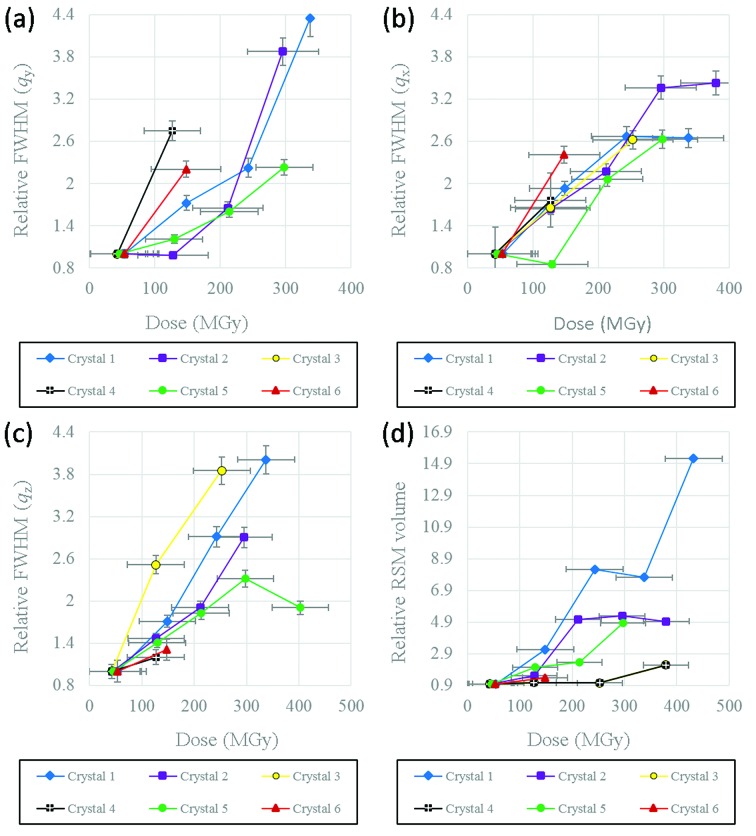
The relative FWHM determined from a Gaussian fit to the experimental RSM data in (*a*) the *q*_*y*_ direction, with initial FWHM values for crystals 1 to 6 of 0.08°, 0.06°, 0.15°, 0.09°, 0.11° and 0.12°, respectively, (*b*) the *q*_*z*_ direction, with initial FWHM values for crystals 1 to 6 of 0.09°, 0.19°, 0.11°, 0.08°, 0.12° and 0.06°, respectively, and (*c*) the *q*_*x*_ direction, with initial FWHM values for crystals 1 to 6 of 0.12°, 0.16°, 0.25°, 0.10°, 0.23° and 0.06°, respectively. (*d*) The relative volume expansion of the 3D RSM calculated as the total number of non-zero counts in the 3D array containing the Bragg reflection with initial RSM volumes for crystals 1 to 6 of 1838, 864, 2569, 3217, 1198 and 1005 µm^3^, respectively. The dose rate determined from *RADDOSE-3D* was 0.42 MGy s^−1^.

The FWHM results from the rocking curve data along with the total volume of the RSM as a function of time are shown in Fig. 4 ▸. In all cases, the width of the rocking curve is observed to increase. Two key factors influencing rocking widths are the unit cell variation, which can occur for example by lattice strain or extended lattice defects, and the size of the crystal. A final factor to consider is that a non-uniform illumination, particularly in conjunction with a dose-dependent lattice change, could lead to broadening of the rocking curve width. One of the major benefits of having access to both reciprocal-space and complex real-space data from the crystals is in the deconvolution of some of these factors.

**Figure 5:**
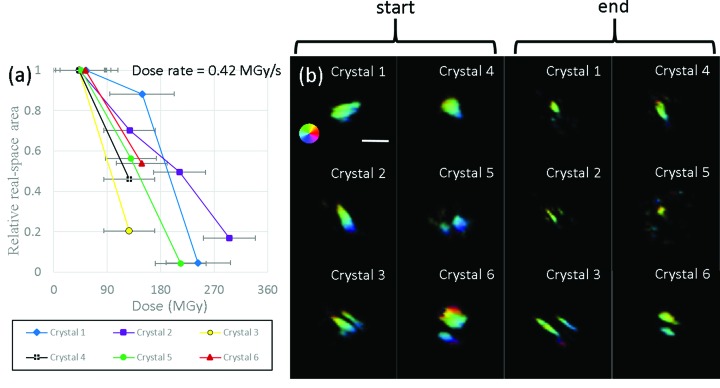
(*a*) Relative change of diffracting area as a function of dose for the six HEWL crystals with initial area values for crystals 1 to 6 of 0.60, 0.48, 0.62, 0.49, 0.49 and 0.90 µm^2^, respectively. (*b*) 2D reconstructions of the crystals at the start and end point of the scan corresponding to the data shown in (*a*). The brightness indicates the amplitude of the reconstruction, whilst the hue indicates the phase (see colour wheel in the starting image of crystal 1). The white scale bar corresponds to 1.1 µm.

To assess the origin of the increase in the FWHM of the rocking curve data, the reconstructed phases of the crystals were also examined (Fig. 5 ▸). In the case of the six crystals studied, the reconstructed phase was slowly varying across the crystal. Between subsequent reconstructions of the same crystal as a function of dose there was little or no variation in this phase structure. This implies that the significant changes observed in the reciprocal-space information are unlikely to be driven by variations induced in the unit cell or an increase in lattice disorder since both these effects should manifest in the phase information. Also the slowly varying phase structure across the reconstructions is a good indication that the incident illumination at the KB focus was relatively uniform. This is consistent with the earlier findings of Huang *et al.* (2012 ▸) who used scanning diffraction measurements of a ZnO crystal to recover the focused illumination profile at the same beamline under similar experimental conditions. It is worth noting that the small phase gradient observed here (particularly at the edges of the crystals) may be an artefact of beam curvature. In terms of the crystal size and shape, however, the reconstructions show that in this case the effect of a non-uniform beam structure is minimal. In all cases, although the phase information does not appear to undergo any significant changes, the apparent size of the crystal is significantly reduced with radiation damage. From this it is concluded that the increase in rocking curve widths and RSM volume which can be tracked as a function of dose (Fig. 4*d* ▸) are likely to be dominated by changes in the apparent size of the diffracting crystal.

Although the data were collected beyond 1000 s, only results for which a reliable FWHM estimate could be obtained are presented. Beyond 1000 s the fluctuation in intensity was too large for a quality Gaussian fit to be performed. Reports in the literature have shown that for both macroscopic and micrometre-sized crystals (Boutet & Robinson, 2006 ▸) the FWHM of the rocking curve increases as a function of dose (Hu *et al.*, 2004 ▸); this trend is also confirmed here. However, it is important to note that very few studies have looked at the variation in FWHM for more than two data points, especially for micrometre-sized crystals. In the previous work, the increase in FWHM has been attributed to a corresponding increase in disorder/mosaicity within the crystal at room temperature (Hu *et al.*, 2004 ▸). One of the primary drivers identified for this reduction in crystal quality has been dehydration which has been observed in room temperature studies.

In the present case, access to real-space images of the crystal during radiation damage *via* BCDI provides a significant advantage. BCDI images allow an assessment to be made of the effect of morphological changes in the diffracting volume of the crystal on the data. The last metric of diffracting crystal area obtained through these coherent imaging studies is discussed in the final results section.

### 3.4. Relative crystal area   

For the work presented here a real-space analysis has been performed of 2D projections of six HEWL crystals during radiation damage and has been compared with the RSM results, in order to draw conclusions about the crystal morphology in three dimensions. The real-space area was calculated as the total area occupied by pixels having an amplitude value greater than 50% of the maximum. When the amplitude within the reconstructed crystal images dropped below 50% of the maximum value, they were considered to be partially disordered and were not included in calculations of the diffracting crystal area. The results of the area analysis are summarized in Fig. 5 ▸.

As with the slightly larger single HEWL micro-crystal previously analysed by Coughlan *et al.* (2015 ▸), for each of the six crystals here there is an overall decrease in diffracting area. Since this area directly contributes to the measured Bragg peak, it can be assumed that regions which apparently ‘switch off’ during radiation damage must become so disordered that they no longer contribute coherently to the measured signal. Given that the crystal is surrounded by a cryo-protectant and held at ∼100 K, it should be emphasized that the reduction in real-space volume contributing to the formation of the diffraction pattern does not mean that there is actual mass loss from the sample. Rather this is indicative of parts of the crystal becoming so disordered that they no longer coherently diffract X-rays and instead just contribute to a diffuse background.

It is also important to note that each 2D reconstruction was performed using data collected at the peak of the rocking curve. Since each scan starts at the beginning of the rocking curve the crystal has already received some dose before the first images are collected. The significant change in the apparent area of the micro-crystal during measurements is an important distinction from studies conducted on macroscopic crystals which tend to show peak broadening due to the formation of crystal mosaics and defects rather than as a result of a change in diffraction volume. For large crystals (hundreds of micrometres or even millimetres across) enclosed within the incident beam the influence of the crystal shape function is small in comparison with the overall crystal quality and mosaicity. In the case of micrometre-sized crystals, changes in the diffracting crystal volume during radiation damage clearly have a significant, even a dominant, influence on the Bragg peak shape and intensity.

In every case examined here, an ever smaller area seems to keep diffracting X-rays whilst the rest of the crystal becomes damaged or destroyed entirely. The BCDI images allow the interior and exterior parts of the crystal at any single dose to be distinguished. However, when trying to compare images from the same crystal measured at different doses the translational invariance of the reconstruction makes spatially correlating the different reconstructions problematic. This means, for example, that caution needs to be applied in drawing conclusions about whether the crystal damage really occurs at the surface. To enable exact placement of coherent diffraction images in terms of their spatial location relative to one another would require a scanning diffraction microscopy approach such as ptychography be used (Peterson *et al.*, 2012 ▸; Vine *et al.*, 2009 ▸). The exact mechanism resulting in some parts of the crystal preferentially suffering the effects of radiation damage is currently not established. However, an explanation for this observation has been developed, as discussed in the next section.

### 3.5. Discussion summary   

In summary, the key/findings observations from the experimental data are:

(1) The integrated intensity data from the single Bragg reflections are very consistent and can be modelled using the exponential decay curve of equation (4). However, to match the experimental data, in the majority of cases Howell’s parameter *H* needed to be adjusted and increased above 10 MGy Å^−1^.

(2) The *d*-spacing varies linearly for lower doses but seems to reach a plateau at higher doses for some crystals (*e.g.* crystals 2, 3 and 4). With the exception of crystal 6, the *d*-spacing always increases compared with the starting value. An assumption often made in the literature is that an increase in *d*-spacing can be directly a result of radiation damage. The *d*-spacing results imply that at lower doses the radiation damage behaviour is approximately linear but that at higher doses there may be a ‘saturation limit’ where the radiation damage behaviour changes.

(3) The FWHM along all three *q*_*x*_, *q*_*y*_ and *q*_*z*_ reciprocal-space vectors as well as the RSM volume increases as a function of dose for all six crystals. Together with the real-space images of the micro-crystals this is taken as strong evidence that the ordered part of the diffracting crystal volume shrinks with increasing dose. Although there are a number of factors that can contribute to rocking curve broadening (unit cell variation, lattice strain and defects, non-uniform illumination *etc*.) the BCDI results suggest that the evolving shape function of the crystal dominates.

(4) From the real-space images, radiation damage appears to preferentially occur in particular regions of the crystal. Looking at the data it is tempting to suggest that the surface of the crystal is damaged *more* quickly than the inner parts; however, complimentary characterization (*e.g.* optical micrographs) is required to confirm this.

A number of studies have highlighted the importance of crystal size in radiation damage at micrometre length scales. For typical crystallography experiments the primary photoelectron kinetic energy results in a mean free path which is generally of the order of 2–3 µm (Ziaja *et al.*, 2001 ▸, 2002 ▸). This mean free path length is normally comparable with or smaller than the diameter of the crystals being measured. In the context of XFEL experiments, it has been argued that radiation-induced damage may be reduced due to the fact that the primary photoelectrons can escape through the crystal surface prior to giving up their energy in initiating secondary damage processes (Caleman *et al.*, 2011 ▸). Similar types of size effects have been observed at the synchrotron where the use of micrometre-sized beams has been shown to result in crystallographic data with a reduced damage signature due to the primary photoelectron ranges being larger than the beam footprint on the sample (Sanishvili *et al.*, 2011 ▸).

In the experiments described here a small, micrometre-sized beam was incident on an even smaller sample. This setup has been less well studied and appears less well understood from a radiation damage perspective than the case of a micrometre-sized beam incident on a larger crystal. The BCDI reconstructions show that the crystal size reduces with dose. If the crystal is shrinking *via* surface damage the process is likely driven by increasing photoelectron escape. In this scenario energy from the primary photoelectrons is deposited outside of the diffracting volume. Since the size of the crystal is even smaller than the beam footprint, the secondary electrons originating from primary events outside of the interaction volume have a reduced chance of depositing their energy inside the crystal. This line of reasoning follows arguments put forth by Sanishvili *et al.* (2011 ▸) and Holton & Frankel (2010 ▸) in which the origin for lower radiation damage rates is explained in the context of micro-focus crystallography experiments performed on larger crystals.

For example, Sanishvili *et al.* studied the effect of the beam size on damage rates in cryo-cooled protein crystals and found that by reducing the beam size from 15.6 µm to 0.84 µm they were able to reduce the radiation damage by a factor of three (Sanishvili *et al.*, 2011 ▸). In their experiment, damage was greatest at the beam center where the highest photo-electronic effect was observed. In the present experiment, the exact opposite behaviour is apparently observed, *e.g.* damage to the surface prior to damage in the centre of the crystal. An important difference between the experiment reported here and that of Sanishvili *et al.* is that the crystals in the present case are fully contained within the beam. Hence the dose received at the edges is not expected to vary as significantly when moving towards the centre as in the Sanishvili *et al.* case. In addition, it is worth noting that, if the current interpretation is correct, the effect observed will become more pronounced as the extremities of the crystal become more disordered and the effective area contributing to the measured diffraction decreases. This will lead to increased primary photoelectron escape from the crystal and a more even dose delivered to the surface compared with the interior of the diffracting crystal.

It is also expected that not all protein crystals will behave in the same way and that radiation damage rates will occur differently depending on the protein system studied. This was clearly identified in a previous study where analysis of a series of diffraction data sets measured from four native as well as four nicotinic acid-soaked crystals of trypsin at 100 K showed a high variability in radiation-sensitivity among individual crystals for both nicotinic acid-soaked and native crystals (Nowak *et al.*, 2009 ▸).

Two factors not discussed so far within this paper are the density of the cryo-protectant and the beam profile. Briefly, it was considered whether the choice of cryo-protectant might influence the radiation damage behaviour of the crystal due to small differences in density between it and the sample itself. For example, if the density of the cryo-protectant is less than that of the crystal, one might expect the ejected photoelectrons to travel a further distance once outside the crystal leading to a reduced amount of radiation damage. To investigate this, the same series of measurements were made on crystals embedded in different cryo-solutions having varying densities. It was found (not shown here) that there was no evidence of a systematic difference in the radiation damage metrics presented here for the different cryo-protectants. This was interpreted as indicating that the differences in density between the cryo-protectant and sample were simply too small to have a measurable influence.

The second factor not taken into consideration here is the beam profile. This has been characterized using knife-edge scans conducted during the experiment and modelling of the beamline optics and it was found that the beam has a Gaussian profile. Also, the beam–sample alignment was confirmed using the combination of X-ray scintillator and microscope to align the beam with the centre of rotation to ensure that the crystal stayed central to the beam during data collection. However, the influence of the beam profile on the damage rates within the crystals cannot be completely eliminated. It may be that, if the beam profile has a strong gradient or if there are hotspots within the profile, this may be the explanation for at least some of the observations during this experiment. From previously published experiments by Huang *et al.* (2012 ▸), it is known that at the KB focus the beam profile resembles a Gaussian. In the present case the sample is comparable with or smaller than the beam FWHM. A follow-up study examining the effect of varying the beam profile and beam size whilst keeping the crystal size constant could help to determine whether, in the present case, these were a significant factor.

## 4. Conclusion   

The results presented here summarize a series of experiments investigating radiation damage in micrometre-sized crystals illuminated with micrometre-sized beams. In these studies the aim has been to shed some light on the key scientific question of whether the radiation damage behaviour observed under these conditions matches the behaviour seen in macroscopic crystals. The coherent imaging and RSM results confirm that the diffracting volume shrinks rapidly with increasing radiation damage. This has a significant effect on the diffraction data which would not be the case with macroscopic crystals. However, it is important to note that the dose in these micro-focus experiments (hundreds of MGy) is much higher than typically used for conventional crystallography (tens of MGy) and from the literature appears much less well understood. For the first time real-space images of micro-crystals undergoing radiation damage can be interpreted. The results from these studies suggest that smaller crystals may have longer lifetimes in micro-focus experiments than would be predicted for macroscopic crystals. The proposed model for this is that the combination of both the beam and the crystal being smaller than the primary photoelectron escape depth leads to an ever-increasing fraction of the cascade energy being deposited in material not contributing to the diffraction signal. This model is able to explain the majority of the observations, but further studies varying beam size and crystal size are required to support or contradict this hypothesis.

One open question which is unresolved is that of a quantitative dose for these micrometre-sized samples. Dose was calculated with details of the physical and chemical properties of the sample used as well as the size and shape of the X-ray beam, but neglecting any effects from photoelectron escape. Though this calculated dose is used in the text, how accurate this estimate actually is for micrometre-sized protein crystals remains uncertain. In addition, the intensity data between crystals is remarkably consistent and can be modelled extremely well using equation (4). However, the usual value of 10 MGy Å^−1^ for Howell’s constant does not, in most cases, yield a good match. Why this discrepancy between the expected and ‘best fit’ values for *H* exists in these experiments remains uncertain at present.

In summary, the results from these combined imaging and reciprocal-space mapping experiments indicate that the global damage behaviour of micro-crystals is different from their macroscale counterparts for the conditions reported here. This discovery, combined with the new insights into coherent imaging metrics, suggests the need for a new and wide-ranging series of studies to investigate the radiation damage behaviours that may be unique to protein micro-crystallography experiments.
